# Modeling of the Kinetics of Supercritical Fluid Extraction of Lipids from Microalgae with Emphasis on Extract Desorption

**DOI:** 10.3390/ma9060423

**Published:** 2016-05-27

**Authors:** Helena Sovová, Beatriz P. Nobre, António Palavra

**Affiliations:** 1 Institute of Chemical Process Fundamentals of the Czech Academy of Sciences, v. v. i., Prague 02101–02117, Czech Republic; 2 Centro de Química Estrutural, Lisbon University, Lisboa 1649-004, Portugal; Beatriz.nobre@tecnico.ulisboa.pt (B.P.N.); antonio.palavra@tecnico.ulisboa.pt (A.P.); 3 Bioenergy Unit, LNEG, Lisbon 1649-004, Portugal

**Keywords:** microalgae, supercritical extraction, oil, kinetics, modeling

## Abstract

Microalgae contain valuable biologically active lipophilic substances such as omega-3 fatty acids and carotenoids. In contrast to the recovery of vegetable oils from seeds, where the extraction with supercritical CO_2_ is used as a mild and selective method, economically viable application of this method on similarly soluble oils from microalgae requires, in most cases, much higher pressure. This paper presents and verifies hypothesis that this difference is caused by high adsorption capacity of microalgae. Under the pressures usually applied in supercritical fluid extraction from plants, microalgae bind a large fraction of the extracted oil, while under extremely high CO_2_ pressures their adsorption capacity diminishes and the extraction rate depends on oil solubility in supercritical CO_2_. A mathematical model for the extraction from microalgae was derived and applied to literature data on the extraction kinetics in order to determine model parameters.

## 1. Introduction

Microalgae are photosynthetic unicellular microorganisms presenting a great genetic variety. More than 50,000 species are supposed to exist, but only about 30,000 have been studied [1]. These microorganisms have not been much used for the production of chemicals, though for some of these, such as some polyunsaturated fatty acids, they are the greatest reserve of the biosphere [2]. Microalgae can produce, in significant amount, lipids similar to vegetable oils, fuels, proteins, essential fatty acids with dietary applications such as linoleic, γ-linolenic, eicosapentaenoic, docosahexaenoic, and arachidonic acids, vitamins (β-carotene, B12 and E), pigments (carotenoids, chlorophylls, phycobiliproteins), waxes, biosurfactants, sterols and other chemical specialties [3].

The lipid content of microalgae can go up to 85% dry weight, though values between 20% and 40% are more typical. The production yield of these compounds relies on the conditions of culture, one of the main factors being the amount of nitrogen to control the lipid content [4].

At the Bioenergy Unit of LNEG (the National Laboratory of Energy and Geology) in Lisbon, several microalgae species are grown with the aim of producing biofuels and high value compounds. Among them are lipids such as alkadienes, carotenoids (astaxanthin, canthaxanthin, lutein, β-carotene) and γ-linolenic acid (GLA). Supercritical fluid extraction of this type of compounds has some advantage over conventional methods, because they can be obtained without contamination by organic solvents and without thermal degradation. Moreover, a high efficiency of extraction can be achieved.

A large number of studies on supercritical CO_2_ (scCO_2_) extraction of lipids and carotenoids from microalgae supplied by the Bioenergy Unit of LNEG have been carried out in the laboratory of the IST Chemical Engineering Department, such as: *Scenedesmus obliquus* [5], *Botryococcus braunii* [6], *Chlorella vulgaris* [7], *Haematococcus pluvialis* [8], *Dunaliella salina* [9], *Spirulina maxima* [10], and *Nannochloropsis* sp. [11], in addition to the research on supercritical fluid extraction of microalgae carried out worldwide. A large amount of experimental data on the effect of extraction conditions on the yield and kinetics of supercritical fluid extraction from microalgae has been published; most of those concerning kinetics of the extraction of oil are cited below.

**Figure 1:**
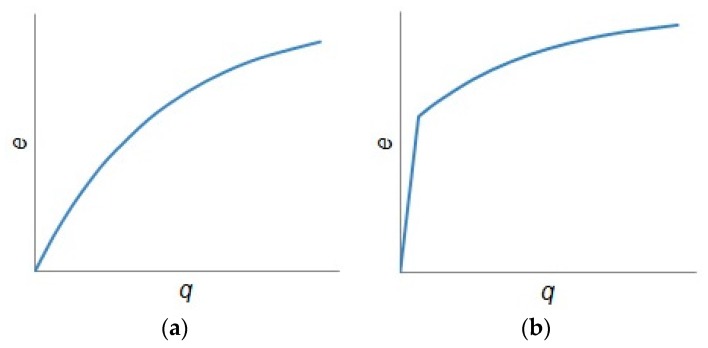
Types of plots of extraction yield against the solvent-to-feed ratio. (**a**) Smooth curve; (**b**) initial straight line and a break to smooth curve.

The situation is rather different in mathematical modelling of the kinetic data. Models for supercritical fluid extraction were applied on experimental data from microalgae only relatively recently. When the extraction yield plotted against extraction time or solvent-to-feed ratio could be matched by a smooth curve (Figure 1a), either a simple equation of first-order process was applied [12,13,14,15], or more complicated models including mass transfer in the pores of solid particles and in the fluid phase [16] and also the solid-fluid partition coefficient [17] were used. When an abrupt decrease in the extraction rate was visible (Figure 1b), the extraction was assumed to be controlled initially by the solute solubility in scCO_2_ and then, after the break of the curve, by internal diffusion [18,19,20].

The objective of this work was to use experimental data on extraction kinetics measured in the IST laboratory as a basis for developing a new mathematical model for scCO_2_ extraction of neutral lipids from microalgae and to verify the model applicability to the data available in the literature. The model assumption on lipid-matrix interaction which decreases the extraction rate and which is suppressed at higher extraction pressures and temperatures seem to be well acceptable.

## 2. Kinetic Data as Basis for the Model

### 2.1. Supercritical Fluid Extraction

The conventional experimental set-up was used in the extraction experiments carried out in both IST and other laboratories which published experimental results analyzed in this work. It consisted of a high pressure pump for CO_2_, the extractor filled with biomass, micrometer valve, and separator. The extractor was filled with dry microalga, pretreated usually by freeze-drying, milling or grinding, and sieving. In some cases, the microalga powder was mixed with inert beads in order to homogenize the extraction bed porosity. After establishing the required pressure and temperature in the extractor, scCO_2_ started flowing with constant velocity through the extraction bed and dissolved oil composed of neutral lipids where triglycerides prevailed. The oil precipitated from the solution after its expansion in the micrometer valve and was collected in the separator. The flow was interrupted in certain intervals in order to gravimetrically measure the increase in extract amount. The amount of passed CO_2_ was determined either after its expansion to ambient pressure by a flowmeter or directly in the high pressure pump. The fatty acid profiles were determined by gas chromatography after methyl esterification of the extract. The details of applied experimental methods are given in the cited literature [5,6,7,8,9,10,11,12,13,14,15,16,17,18,19,20].

### 2.2. Preliminary Analysis of IST Experimental Data on Extraction Kinetics

#### 2.2.1. Phase Equilibrium

**Figure 2:**
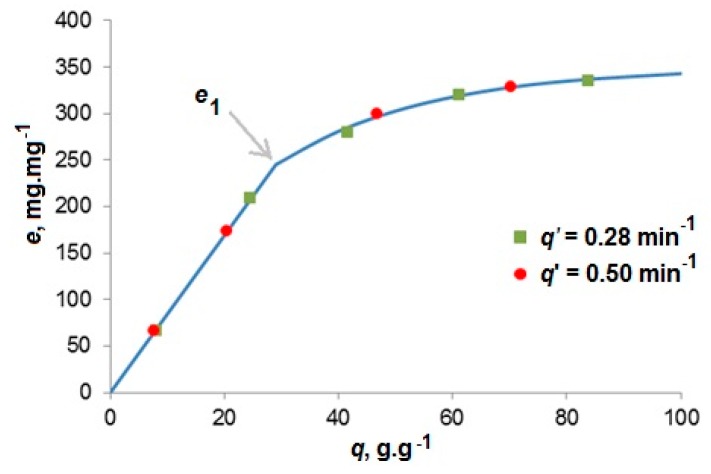
Yield of oil from *Nannochloropsis* sp. [11] at 30 MPa and 40 °C. The break of the curve is at the extraction yield *e*_1_. The specific flow rate is inversely proportional to the residence time.

The experiment carried out by Nobre *et al.* [11] enabled us to estimate the equilibrium fluid phase concentration of extracted lipids. Two extraction runs were carried out with two different CO_2_ flow rates, 0.35 and 0.62 g·min^−1^, and with the feed 1.25 g of thoroughly ground *Nannochloropsis* sp. When plotted against the solvent-to-feed ratio, the extraction yields from both runs lay on a single curve not only in the first, linear section, but also when the extraction slows down (Figure 2). This proves that the solution flowing out of the extractor under the conditions of slow flow rates was practically saturated. As the fluid phase concentration given in mass of extract over the mass of CO_2_ is equal to the slope of extraction curve *e*(*q*) connecting the experimental points, local slopes of the overlapping extraction curves indicate fluid phase equilibrium concentrations not only in the first, linear section of extraction curve, but also in the second, curved section.

In accordance with the interpretation of overlapping extraction curves made by Perrut *et al.* [21] who measured the yield of oil from sunflower seeds, the slope of the first, linear section indicates the oil solubility in scCO_2_ and the second section of the curve, which begins at extraction yield *e*_1_, is related to oil desorption from microalgae matrix. The initial slope was really in the range of estimated oil solubility in scCO_2_.

The asymptotic yield of oil was 350 g·kg^−1^. It is less than the oil content in microalgae, 407 g·kg^−1^, determined by Soxhlet extraction with *n*-hexane. Thus, about 14% of the oil could not be extracted. (n-Hexane as a non-polar solvent dissolves same substances as CO_2_). We assume that hot *n*-hexane is able to make the cell walls permeable, in contrast to scCO_2_. Our hypothesis is that the cells containing 14% of oil remained intact after the grinding of dry biomass in a ball mill and the oil from intact cells could not pass through impermeable cell walls.

#### 2.2.2. Pressure Dependence of Phase Equilibrium

**Figure 3:**
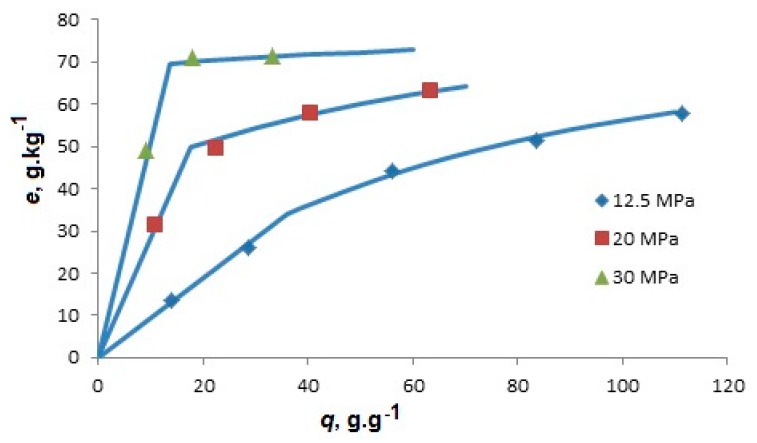
Yield of extracellular hydrocarbons from *Botryococcus braunii* [6] at 40 °C and *q*’ = 0.39 g·min^−1^. The feed was *N* = 2 g.

Microalga *Botryococcus braunii* [6] was extracted with scCO_2_ at specific flow rate *q*’ = 0.39 g·min^−1^, temperature 40 °C, and pressures 12.5, 20.0, and 30.0 MPa. The strain chosen for the study produced hydrocarbons which were accumulated on the outer side of cell walls. Their content in the alga was 76 g·kg^−1^ and their major components were dienes C31, C29, and C37, and triene C29. Before the extraction, freeze-dried alga was slightly crushed in a mortar to separate larger cell aggregates. As the only mass transfer resistance for hydrocarbons was located in supercritical fluid between the cells forming aggregates and boundary layer around the aggregates, and the specific flow rate was comparable to that applied in the extraction of *Nannochloropsis* sp. [11], the extraction was assumed to be controlled by phase equilibrium. Figure 3 shows two effects of increase in pressure: not only that the lipid solubility in scCO_2_ increases, which is evident from the slope of the initial straight section of extraction curve, but the break of the curve appears at higher extraction yield and the amount of slower extracted hydrocarbons decreases, which corresponds to a decreased adsorption capacity of microalga.

#### 2.2.3. Effect of Microalgae Pretreatment

**Figure 4:**
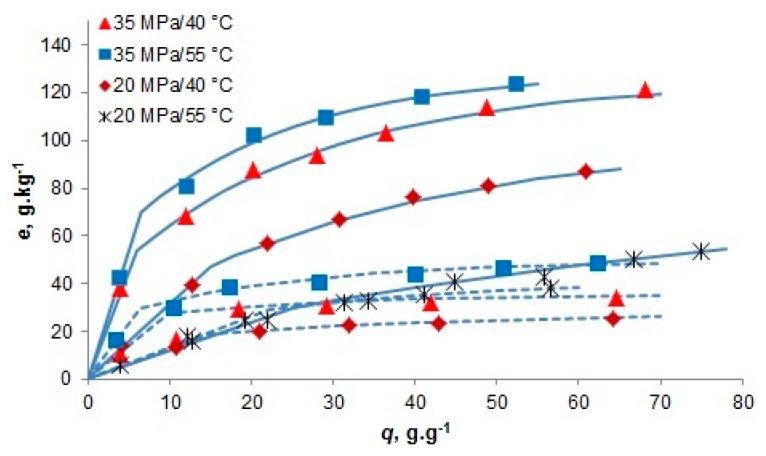
Oil extraction from *Chlorella vulgaris* [7] with scCO_2_ at q’ = 0.16 min^−1^. The feed was *N* = 5 g. Full lines: crushed alga, dashed lines: whole alga.

In another study [7], freeze-dried samples of the microalga *Chlorella vulgaris* containing 185 g·kg^−1^ oil (determined by *n*-hexane extraction), either crushed or without any other pretreatment were subjected to scCO_2_ extraction. As shown in Figure 4, the estimated asymptotic yield of oil increased after crushing from 15%–27% to 50%–70%, related to the yield obtained with *n*-hexane.

We can assume that the asymptotic yield estimated from experimental runs carried out at the most severe extraction conditions 35 MPa and 55 °C, which is 27% without crushing and 70% for the crushed algae, approximately corresponds to the percentage of open cells in each sample: approximately 27% of cell walls were open already by freeze drying, about 43% by crushing, and 30% of cells remained intact even after the pretreatment.

The solubility of lipids in scCO_2_ depends on their chemical composition, besides pressure and temperature. When C16 fatty acids prevail in triglycerides, the solubility of extracted oil is higher than the solubility of vegetable oils with prevailing C18 fatty acids, for which a correlation with extraction temperature and solvent density was published by del Valle *et al.* [22]. The main constituents of the *C. vulgaris* fatty acid profile were Cl8:1, C16:0, and Cl8:3 fatty acids with weight fractions 0.41, 0.22, and 0.09, respectively. As the fatty acids with 18 carbons prevail, the oil solubility in scCO_2_ can be expected to be similar to that of common vegetable oils, where also C18 fatty acids prevail. The data in Table 1 show that the fluid phase concentration in the first extraction period was very similar to the average value of vegetable oil solubility in scCO_2_, when the aggregates of dry biomass were crushed. However, in the case of whole particles, the mass transfer resistance was large enough to hinder the extraction rate substantially even at a specific flow rate as low as 0.16 min^−1^.

## 3. General Hypothesis

According to the above analysis of experimental data, we can assume that at least a part of lipids is bound to matrix and is desorbed in the course of extraction. The second assumption is based on the fact that the asymptotic extraction yield depends on the extent of cell walls distortion by the pretreatment. The hypothesis is that the solute is extracted only from the cells opened by the pretreatment; both open and closed cells form agglomerated particles and the solute from open cells is first transferred to the solvent which fills the space between the cells, diffuses to the particle surface, and then to bulk fluid. Thus, the model for scCO_2_ extraction of oil from microalgae should include adjustable content of accessible solute, *x_u_*, characteristic timer of mass transfer resistance dependent on microalgae pretreatment and extraction conditions, *t_c_*, and parameters of phase equilibrium: solubility, *y_s_*, adsorption capacity, *x_t_*, and partition coefficient, *K*.

## 4. Results

### 4.1. Model for Oil Extraction from Microalgae

#### 4.1.1. Model Equations

Mass balance equations are written for the extraction from a packed bed represented by a series of *n* mixers of equal size:

(1) $$\frac{dy_{j}}{dt}=n\frac{y_{j-1}-y_{j}}{t_{r}}+\frac{y_{j}^{+}-y_{j}}{t_{c}};\;\frac{dx_{j}}{dt}=-\gamma \frac{y_{j}^{+}-y_{j}}{t_{c}}\text{ for }j=1,2,\;...,n$$

(2) $$e=q\prime \int_{0}^{t}y_{n}dt$$

The driving force is the difference between the fluid phase concentration at the interface with liquid lipids in opened cells, *y_j_*^+^, and bulk fluid concentration, *y_j_*. The characteristic mass transfer resistance time, *t_c_*, is inversely proportional to the mass transfer coefficient, and *t_r_* is the residence time. The initial and boundary conditions are

*t* = 0: *e* = 0, *y_j_* = *y*_0_, *x_j_* = *x_u_* − <*y*_0_ for *j* = 1, 2, …, *n*; *j* = 1: *y*_j − 1_ = 0 (3)

Phase equilibrium is defined according to Perrut *et al.* [21]:

*y*^+^ = *y_s_* for *x* ≥ *x_t_*; *y*^+^ = *Kx* for *x* < *x_t_*; *K* < *y_s_*/*x_t_* (4)

The model assumes that as long as the solid phase concentration is higher than the adsorption capacity *x_t_*, the extracted solute is freely dissolved and the equilibrium fluid phase concentration is equal to the solubility *y_s_*. Below *x_t_*, all solute remaining in the solid phase is adsorbed on the matrix and phase equilibrium is described by adsorption isotherm with partition coefficient *K*.

Besides the parameters characterizing phase equilibrium, the model contains the mass transfer resistance *t_c_*. As the microalgae cells form agglomerated particles, the solute from open cells first dissolves in the solvent filling the space between the cells, diffuses to particle surface and further to bulk fluid. Thus, the characteristic time for internal diffusion, *t_i_*, is added to the fluid phase mass transfer characteristic time, *t_f_*, to give the overall characteristic mass transfer time *t_c_*.

The extraction bed is characterized by particle size and void fraction, which enables calculation of CO_2_ amount between particles and its ratio to microalga feed, γ. The extraction time *t* as independent variable in the above equations can easily be converted to the frequently used solvent-to-feed ratio, taking into account that *q* = *q*’*t*.

The number of mixers allows us to simulate different extent of axial dispersion, from an ideal mixer (lumped parameter model) with *n* = 1 to plug flow with *n* → ∞. The model properties will be inspected for both limiting flow patterns.

#### 4.1.2. Plug Flow

The mass balance is given by partial differential equations

(5) $$\frac{\partial y}{\partial t}=-\frac{1}{t_{r}}\frac{\partial y}{\partial z}+\frac{y^{+}-y}{t_{c}};\;\frac{\partial x}{\partial t}=-\gamma \frac{y^{+}-y}{t_{c}}$$

(6) $$e=q\prime \int_{0}^{t}y\left(z=1\right)dt$$

As long as free solute is dissolved, *y*^+^ = *y_s_*, the extraction yield obtained by integration of Equations (5) and (6) is directly proportional to extraction time:

(7) $$y=y_{s}\left[1-exp\left(-z\frac{t_{r}}{t_{c}}\right)\right];\;x=x_{0}-\gamma y_{s}\frac{t}{t_{c}}exp\left(-z\frac{t_{r}}{t_{c}}\right);\;e=y_{s}\left[1-exp\left(-\frac{t_{r}}{t_{c}}\right)\right]q\prime t$$

(A short initial time for establishing concentration profiles in the extractor was neglected.) The switch to desorption at *x* = *x_t_* occurs gradually along the axial coordinate in accordance with axial concentration profile *x*(*z*). The plug flow model equations for desorption must therefore be solved numerically.

Poletto and Reverchon [23] and Cocero and García [24] analyzed the model equations for the case when desorption starts from the very beginning of extraction, *x*_0_ < *x_t_*. The solution for the fluid phase concentration at the extractor outlet published in these papers is in the Laplace domain and needs transformation to the time domain. Poletto and Reverchon [23] further analyzed the numerically calculated yield curves *e*(*t*) and found that they approach two limiting cases for low and high values of *t_c_*/*t_r_*. When *t_c_*/*t_r_* < 0.02, the mass transfer resistance is practically negligible and the extraction yield does not depend on extraction time but on the solvent-to-feed ratio, *q*:

(8) $$e=Kx_{0}q\prime t=Kx_{0}q$$

When *t_c_*/*t_r_* > 2, flow pattern has little effect on the extraction yield and extraction curve *e*(*t*) can therefore be calculated according to a lumped parameter model (model for ideal mixer) with ordinary differential equations. Again, the solution for the lumped parameter model which was published is in the Laplace domain [23]. For *t_c_*/*t_r_* values between these limits, the plug flow model should be solved numerically.

#### 4.1.3. Ideal Mixer

The model equations are identical with Equations (1)–(4) where *n* = 1 is substituted. Up to three periods can be distinguished in the course of extraction according to this model: dissolution of free solute, washing without mass transfer, and desorption of solute from microalga matrix.

Extraction of free solute. As long as *x* > *x_t_*, *y*^+^ = *y_s_* and the exact solution of the mass balance equation for fluid phase is

(9) $$y=\frac{t_{r}}{t_{r}+t_{c}}y_{s}+\left(y_{0}-\frac{t_{r}}{t_{r}+t_{c}}y_{s}\right)exp\left(-\frac{t_{r}+t_{c}}{t_{c}}\frac{t}{t_{r}}\right)$$

However, as the effect of initial conditions quickly disappears, we shall assume for the sake of simplicity that

(10) $$y=\frac{t_{r}}{t_{r}+t_{c}}y_{s}\text{ for }x>x_{t}$$

from the beginning of dynamic extraction at *t* = 0. The other variables in this extraction period are

(11) $$x=x_{u}-\gamma y_{s}\frac{t_{r}+t}{t_{r}+t_{c}};\;e=\frac{y_{s}}{1+\frac{t_{c}}{t_{r}}}q\prime t$$

The period of constant extraction rate ends at *t* = *t*_1_ when the solid phase concentration falls to *x_t_*:

(12) $$t_{1}=\frac{x_{u}-x_{t}}{\gamma y_{s}}\;\left(t_{r}+t_{c}\right)-t_{r};\;y_{1}=\frac{t_{r}}{t_{r}+t_{c}}y_{s};\;x_{1}=x_{t};\;e_{1}=x_{u}-x_{t}-\gamma y_{1}$$

Convection without mass transfer. If *y*_1_ > *Kx_t_*, the solute is washed out of the mixer at fixed *x* = *x_t_*:

(13) $$y=y_{1}exp\left(-\frac{t-t_{1}}{t_{r}}\right);\;e=e_{1}+\gamma y_{1}\left[1-exp\left(-\frac{t-t_{1}}{t_{r}}\right)\right]$$

until the bulk fluid phase concentration decreases to *Kx_t_* at *t* = *t*_2_ when solute desorption from plant matrix starts:

(14) $$t_{2}=t_{1}+t_{r}ln\left(\frac{y_{1}}{Kx_{t}}\right);\;y_{2}=Kx_{t};\;x_{2}=x_{t};\;e_{2}=e_{1}+\gamma \left(y_{1}-Kx_{t}\right)$$

If *y*_1_ ≤ *Kx_t_*, the period of washing is skipped and desorption period follows directly after the extraction of free solute. In such case, *t*_1_, *y*_1_, and *e*_1_ from Equation (12) must be substituted for *t*_2_, *y*_2_, and *e*_2_, respectively, in the following equations of the desorption model.

Desorption. Desorption from the microalgae matrix takes place in the last extraction period which begins at *t* = *t*_2_. As mentioned above, Poletto and Reverchon [22] offered a solution for the lumped parameter desorption model in the Laplace domain. A more detailed model, simulating besides the external mass transfer also diffusion in pores of spherical particles, was formulated and solved by Peker *et al.* [25] and by Goto *et al.* [26] for particular initial conditions. We have derived the solution of the lumped parameter desorption model with mass transfer resistance for arbitrary initial concentrations (see Appendix). The sum of the yield *e*_2_ and the yield calculated according to the desorption model (Equation (A7) in Appendix) with initial conditions *x* = *x*_2_, *y* = *x*_2_, *t* = *t*_2_ is:

(15) $$\begin{array}{c} y=\frac{1}{R}\left(K\frac{x_{2}+\gamma y_{2}}{t_{c}}A+y_{2}B\right);\;x=\frac{1}{R}\left[\left(\frac{x_{2}}{t_{r}}+\frac{x_{2}+\gamma y_{2}}{t_{c}}\right)A+x_{2}B\right]\\ e=e_{2}+\left(x_{2}+\gamma y_{2}\right)\left[1-\frac{B}{R}-\frac{A}{R}\frac{1+\gamma K}{t_{c}}\right]-\frac{A}{R}\frac{x_{2}}{t_{r}} \end{array}$$

where

(16) $$\begin{array}{c} A=\exp\left[p_{1}\left(t-t_{2}\right)\right]-\exp\left[p_{2}\left(t-t_{2}\right)\right];B=p_{1}\exp\left[p_{1}\left(t-t_{2}\right)\right]-p_{2}exp\left[p_{2}\left(t-t_{2}\right)\right]\\ p_{1}=\frac{-S+R}{2},\;p_{2}=\frac{-S-R}{2},\;S=\frac{1}{t_{r}}+\frac{1+\gamma K}{t_{c}},\;R=\sqrt{S^{2}-4\frac{\gamma K}{t_{r}t_{c}}} \end{array}$$

Extraction without constant rate period. When the initial concentrations are *x*_0_ = *x_t_*, *Kx_t_* < *y*_0_ ≤ *y_s_*, the extraction starts with the washing period. Therefore, *t*_1_ = 0, *y*_1_ = *y*_0_, and *e*_1_ = 0 has to be substituted in Equations (13) and (14). When *x*_0_ < *x_t_*, the extraction process consists only of desorption period. Then, *t*_2_ = 0, *x*_2_ = *x*_0_, *y*_2_ = *y*_0_, and *e*_2_ = 0 is substituted in Equations (15) and (16).

**Figure 5:**
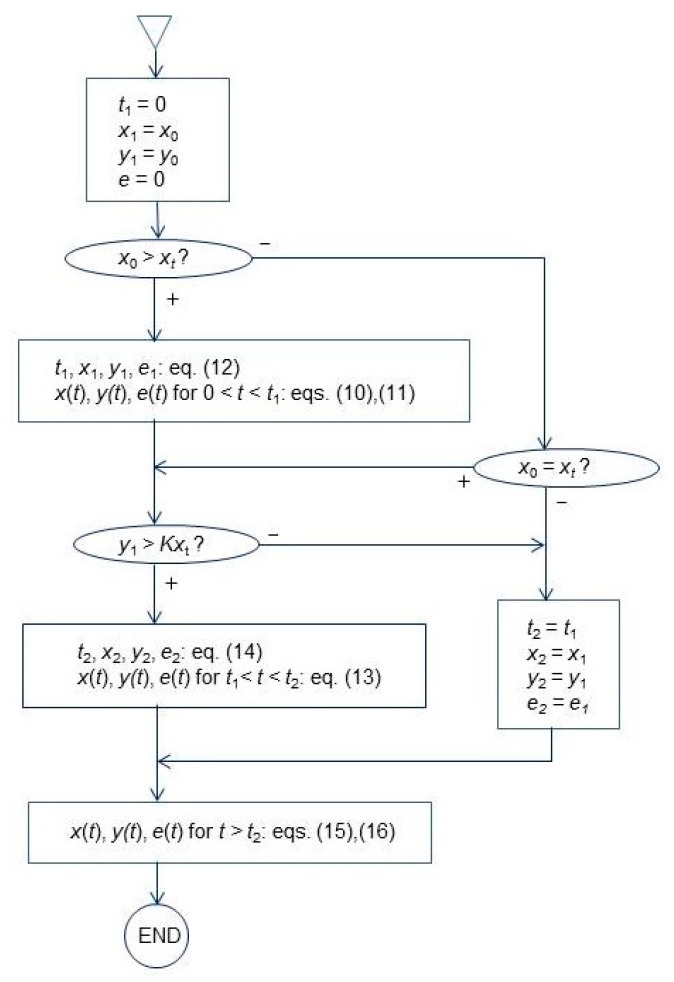
Algorithm of the lumped parameter model solution.

The scheme of model integration is shown in Figure 5. Logically, the initial fluid concentration *y*_0_ may not be higher than the equilibrium concentration:

(17) $$0\leq y_{0}\leq y_{s}\text{ for }x_{0}\geq x_{t};\;0\leq y_{0}\leq Kx_{t}\text{ for }x_{0}<x_{t}$$

#### 4.1.4. Interaction of Model Parameters

As the extraction yield is simultaneously affected by phase equilibrium, mass transfer resistance, and flow pattern, all model parameters cannot be uniquely determined from a single comparison of calculated extraction curve *e*(*t*) with experimental data. If the extraction begins by dissolution of free solute, adsorption capacity can be estimated from the point where the slope of the extraction curve suddenly decreases; however, the other parameters interfere. The initial slope of extraction curve in the period of extraction of free solute depends on both solubility *y_s_* and mass transfer resistance *t_c_*, except for residence times much larger than mass transfer resistance. Thus, if the value of parameter *t_c_* is changed from *t_ca_* to *t_cb_* and the initial slope of extraction curve is fixed by simulated experimental data, the value of parameter *y_s_* should be shifted from *y_sa_* to *y_sb_*. In the case of plug flow, the relationship between the adjusted *t_c_* and *y_s_* values follows from Equation (7):

(18) $$\frac{y_{sb}}{y_{sa}}=\frac{1-exp\left(-\frac{t_{r}}{t_{ca}}\right)}{1-exp\left(-\frac{t_{r}}{t_{cb}}\right)}$$

Similarly, the relationship between *y_s_* and *t_c_* in the mixer is derived from Equation (11):

(19) $$\frac{y_{sb}}{y_{sa}}=\frac{t_{r}+t_{cb}}{t_{r}+t_{ca}}$$

The interaction between desorption model parameters *t_c_* and *K* is derived from the approximate relationship for time constant *T*_1_ given by Equation (A10) in Appendix. If the parameters are changed from *t_ca_* to *t_cb_* and from *K_a_* to *K_b_*, and the time constant should remain unchanged, the relationship between the parameter values is

(20) $$\frac{t_{cb}}{t_{r}}=\frac{t_{ca}}{t_{r}}\frac{K_{b}}{K_{a}}+\frac{K_{b}}{K_{a}}-1$$

Thus, if the solubility of extracted substance in scCO_2_, *y_s_*, is known and if the degree of axial mixing is estimated, the characteristic time of mass transfer *t_c_* can be estimated from the slope of initial straight section of extraction curve. The value of partition coefficient, *K*, is then determined from the curved part of extraction curve, because the value of *t_c_* is known.

It should be taken into account that the partition coefficient appears in equations for extraction yield always in the product γ*K*. If the mass of CO_2_ in the space between plant particles is not measured or estimated correctly, the value of *K* evaluated by fitting the calculated extraction curve to experimental data will be biased.

### 4.2. Comparison of Model with Experimental Data

The effects of flow rate, Q’, and particle size, *d_p_*, on the fluid phase mass transfer coefficient and thus on the external mass transfer resistance *t_f_* can be estimated from the correlation [27]

(21) $$Sh=0.206Re^{0.8}Sc^{0.33}$$

After substitution for individual terms in the equation we obtain

(22) $$t_{f}=\frac{\varepsilon }{k_{f}a_{0}}=\frac{d_{p}^{1.2}}{1.236}\frac{\varepsilon }{1-\varepsilon }\frac{\mu^{0.47}\rho_{f}^{0.33}}{D_{12}^{0.67}}{\left(\frac{Q^{\prime }}{S_{E}}\right)}^{-0.8}$$

For finely ground microalgae particles, we can neglect the mass transfer resistance in particle pores and expect a direct proportionality of *t_c_* and *d_p_*^1.2^ and an indirect proportionality of *t_c_* and *Q*’^0.8^.

When the residence time *t_r_* is sufficiently large in comparison with *t_c_*, the effect of mass transfer resistance on the extraction yield is suppressed and flow pattern affects the yield to a large extent. On the other hand, when *t_c_*/*t_r_* is sufficiently large, the extraction yield does not depend on flow pattern (as shown also by Poletto and Reverchon [23]) but it depends strongly on *t_c_*. The solubility of lipids in scCO_2_ depends on their chemical composition, besides pressure and temperature. When C16 fatty acids prevail in triglycerides, the solubility of extracted oil is higher than the solubility of vegetable oils with prevailing C18 fatty acids, for which a correlation with extraction temperature and solvent density was published by del Valle *et al.* [27].

The density of particles was assumed to be ρ*_s_* = 950 kg.m^−3^ and for the density of scCO_2_ the NIST database was used [28]. If the particles of alga were mixed with glass beads, the bed characteristics were assumed to be ε = 0.40 and γ = ρ*_f_*ε*V*/*N*; the volume of extraction bed, if not specified in the paper, was estimated from the volumes of alga particles and glass beads (whose density is approximately 2.5.10^3^ kg·m^−3^) and from the void fraction. If the extraction bed consisted from alga particles without glass beads, the void fraction was assumed to be ε = 0.35 and γ was calculated as ερ*_f_*/((1 − ε) ρ*_s_*).

#### 4.2.1. Application of Lumped Parameter Model

**Figure 6:**
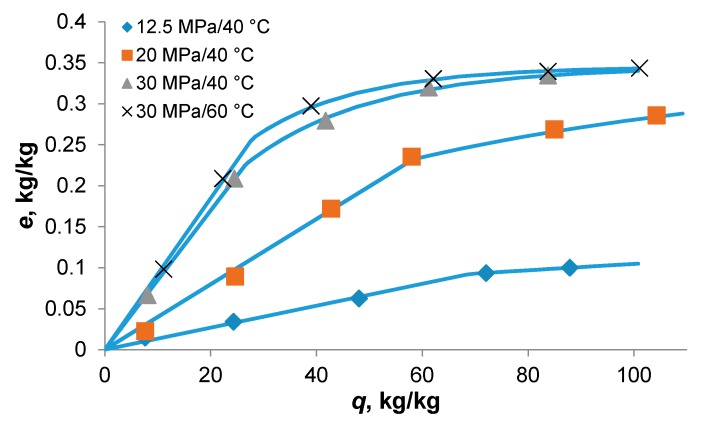
Extraction yield from *Nannochloropsis* sp. [11]: the symbols represent experimental data and the extraction curves were calculated with the lumped parameter model.

For short extractors, we used the model of ideal mixer with analytical solution. It was applied to the extraction of oil from *Nannochloropsis* species [11] carried out in a 5 cm^3^ extractor filled with 1.25 g alga and glass beads. To simulate the overlapping extraction curves measured at 30 MPa, 40 °C with *Q*’ = 0.35 g·min^−1^ and 0.62 g·min^−1^, we assumed in accordance with Equation (22) that *t_c_* for the higher flow rate was 1.6 times lower than the *t_c_* for the lower flow rate because the internal mass transfer resistance in the finely ground alga was negligible. By fitting the calculated extraction curves to experimental data (see Figure 6) the model parameters listed in Table 1 were determined. As *t_c_* could be estimated only from two experimental runs at different flow rates at 30 MPa and 40 °C, the same value was of *t_c_* was used for the other extraction conditions. The adsorption capacity decreased and partition coefficient *K* and solubility *y_s_* increased with increasing pressure and temperature.

The lumped parameter model was fitted also to the kinetic data on extraction of hydrocarbons from *Botryococcus braunii* [6] with alga feed 2 g. The hydrocarbons were extracted in a mixture with triglycerides, occupying thus a part of the solvent capacity. The particles were larger than in the previous extraction because alga was only slightly crashed. Thus, a larger *t_c_* than previously was assumed; its value was fixed at 1 min (see Table 2). Though *t_c_* interferes with two adjusted equilibrium parameters, *ys* and *K*, the trends of equilibrium parameters with increasing pressure would not be changed if the value of *t_c_* is shifted: *y_s_* and *K* increase, while *x_t_* decreases, like in the extraction of *Nannochloropsis* sp. [11]. The decreasing adsorption capacity with increasing pressure is observed not only in the extraction of microalgae. Xing *et al.* [29], who studied the adsorption of artemisinin dissolved in scCO_2_ on silica gel, related the adsorbed amount of artemisinin to the solvating power of CO_2_. They hypothesized that the solvent competes with artemisinin for adsorption sites on silica gel and its competitive adsorption enhances as pressure increases.

We tried to adjust the model also to the data on extraction of oil from *Scenedesmus obliquus* [20] with a feed of 0.5 g. This experiment differs from other extraction experiments modelled in this paper by use of ethanol in 5% concentration as CO_2_ modifier and by extremely short residence time. We were not able to estimate the solubility with sufficient accuracy and therefore we could not evaluate *t_c_* and *K*. The adsorption capacity *x_t_* between 0.08 and 0.13 kg·kg^−1^ was only slightly dependent on extraction pressure and temperature in the range 15–30 MPa and 45–65 °C, respectively. One phenomenon contradicting the model assumption was observed: all experimental runs could be modelled with *x_u_* = 0.208 kg·kg^−1^ except for the one conducted at maximum pressure and temperature where the estimated content of extractable oil in alga increased to *x_u_* = 0.290 kg·kg^−1^. Very probably, the cells which remained closed after the grinding of dry microalga were opened when exposed to modified CO_2_ at 30 MPa and 65 °C.

#### 4.2.2. Application of Model with Series of Mixers

For higher extraction beds we integrated model Equations (1)–(4). Because of the discontinuity in the equilibrium relationship for equilibrium we applied a simple Eulerian integration method with sufficiently short increments of time. The initial number of mixers was selected *n* = 5 and if the calculated curve did not fit the experimental yields, *n* was adjusted as necessary.

Andrich *et al.* extracted *Nannochloropsis* sp. [13] and *Spirulina platensis* [14] at two temperatures, 40 and 55 °C, and at pressures increasing up to 70 MPa. The lyophilized and ground alga was sieved to maximum particle size 0.35 mm and mixed with glass beads. The adjusted values of *x_u_* were 0.255 kg·kg^−1^ for *Nannochloropsis* sp. and 0.078 kg·kg^−1^ for *S. platensis*. The solubility of oils (see Table 3) was assumed to be equal to that of vegetable oil with prevailing C18 fatty acids. The important result is that the mass transfer resistance increases with increasing pressure, it is with increasing distance from CO_2_ critical point.

The data measured by Mendes *et al.* [7] for lipid extraction from *Chlorella vulgaris* were simulated with the model using parameter values listed in Table 4. The calculated extraction curves were very similar to those obtained in preliminary evaluation shown in the Section 2.2.3, but, in contrast to the preliminary evaluation, the amount of extractable lipids was fixed and the final extraction yield was fitted by adjusting the partition coefficient. The particle size affected mostly the mass transfer resistance *t_c_* which was decreased by crushing of alga by order of magnitude. The adsorption capacity of whole particles was lower than the capacity of crushed alga.

**Figure 7:**
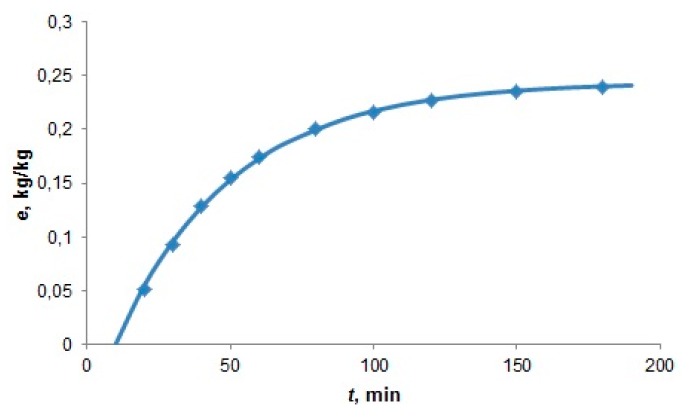
Extraction yield from *Chlorella protothecoides* [17] (*N* = 1 g, *t_r_* = 34 min). Model parameters: *x_u_* = 0.244 kg·kg^−1^, *K* = 0.20 kg·kg^−1^, *n* = 1.

Chen and Walker [17] adjusted the residence time in the extraction of *Chlorella protothecoides* to approximately 34 min, using the feed of 1 g of finely ground biomass (*d_p_* ≤ 0.25 mm) mixed with 90 g of glass beads and the flow rate 0.73 g·min^−1^. The space above the layer of microalga mixed with glass beads was filled with another layer of glass beads which was the main reason for transport delay indicated in Figure 7 by 10 min shift. The extraction pressure and temperature were 35 MPa and 50 °C, respectively. The mass transfer resistance is supposed to be smaller than 1 min under these conditions, and even up to *t_c_* = 10 min it would have no effect on the calculated extraction yield. All extractable lipids in the microalga were adsorbed on matrix (see Figure 4). It is interesting that the shape of extraction curve was best simulated with *n* = 1, though the flow pattern through a bed of spheres is expected to be close to plug flow. Possibly, the high degree of mixing corresponds to natural convection which could develop at extremely low interstitial velocity.

The paper of Mouahid *et al.* [18] on the extraction of lipids at 40 MPa and 60 °C does not contain the values of feed, which could be however derived for *Cylindrotheca closterium* from its extraction curves given simultaneously as *e*(*t*) and *e*(*q*). On the other hand, the values Q’ = 0.41 kg·h^−1^, *x_u_* = 0.135 kg·kg^−1^ and *y_s_* = 0.00155 kg·kg^−1^ were specified [18]. The solubility was set equal to the average of initial slopes of extraction curves e(q), not taking into account the effect of particle size on the mass transfer rate. It is evident from comparison with CO_2_ solubility of vegetable oils at 40 MPa and 60 °C, 0.0151 kg·kg^−1^ [22], that it is too low, and therefore we substituted in our model *y_s_* = 0.0151 kg·kg^−1^. The residence time estimated from the feed varied between 0.15 and 0.6 min. Using these values in the calculation of the extraction curve, the partition coefficient was adjusted to *K* = 0.04 kg·kg^−1^ for particles smaller than 0.16 mm and *K* = 0.03 kg·kg^−1^ for particles larger than 1 mm. The mass transfer resistance was *t_c_* = 1.1 min for particles smaller than 0.16 mm and *t_c_* = 9 min for particles larger than 1 mm. Thus, the effect of particle size on *t_c_* was similar as in the experiment of Mendes *et al.* [7]. The adsorption capacity of the particles smaller than 0.16 mm increased from *x_t_* = 0.06 kg·kg^−1^ for the biomass dried by air flow to *x_t_* = 0.08 kg·kg^−1^ for the freeze-dried alga. The adsorption capacity of the particles larger than 1 mm dried by air flow was estimated as *x_t_* = 0.087 kg·kg^−1^. The effect of flow pattern (number of mixers) on extraction yield was negligible with residence times as short as in this experiment.

Finally, the extraction yields published by Crampon *et al.* [30] who examined the effect of drying and particle size on extraction yield from *Nannochloropsis oculata* were modelled. As C16 fatty acids prevailed to a large extent in the fatty acid profile of alga, we calculated the solubility in scCO_2_ by the Chrastil equation, which was fitted to the data on tripalmitin solubility in scCO_2_ [31]. The number of mixers (*n* = 5) was fixed. The paper contains data on the extraction with alga feed ranging from 10 g up to 15 kg. We estimate the residence time in two modelled experiments as 0.6 min for the feed 10 g and 3.9 min for the feed 2 kg. The first pair of results in Table 5 shows the effect of particle size on extraction yield; the only model parameter to be changed was the content of accessible lipids in alga. The extraction from alga dried under air flow was faster than the extraction from freeze-dried alga; the parameters of the model adjusted to the experimental data differed mainly by adsorption capacity. This result suggests that almost all adsorption sites were damaged by air-drying, in contrast to freeze-drying. The effect of particle size on extraction yield was examined also in the large scale extraction with 2 kg feed and with particle size 0.5 and 2 mm. For the larger particles, the mass transfer resistance was comparable with that observed in the extraction from whole particles of *C. vulgaris* listed in Table 3.

## 5. Comparison with BIC Model

The broken and intact cell model was used in previously published studies [18,19,20] to evaluate extraction curves of type b in Figure 1. We can compare both models by modeling a simple case of extractor as ideal mixer, *n* = 1. The extraction curve according to the BIC model is approximated by equation

(23) $$e=\frac{qy_{s}}{1+\frac{t_{f}}{t_{r}}}\text{ for }q\;\leq \;q_{1},\;e\;\leq \;e_{1};\;e=x_{u}-\left(x_{u}-e_{1}\right)exp\left(-\frac{q-q_{1}}{q\prime t_{i}}\right)\text{ for }q>q_{1},\;e>\;e_{1}$$

while for the extraction of microalgae we can use an approximate expression

(24) $$e=\frac{qy_{s}}{1+\frac{t_{c}}{t_{r}}}\text{ for }q\;\leq \;q_{1},\;e\;\leq \;e_{1};\;e=x_{u}-\left(x_{u}-e_{1}\right)exp\left(-\frac{q-q_{1}}{T_{1}q^{\prime }}\right)\text{ for }q\;>q_{1},\;e\;>\;e_{1}$$

The first, linear part of the extraction curve is identical for both models except for the external mass transfer resistance *t_f_* in the BIC model, which assumes initially the extraction from particle surface while the model for microalgae extraction assumes the extraction from the cells inside particles with combined mass transfer resistance *t_c_* from the very beginning.

The point (*q*_1_, *e*_1_) represents in the BIC model the moment when the oil at particle surface is exhausted and the extraction from the cells inside particles begins. In the model for microalgae extraction, the free oil is exhausted and the extraction of oil adsorbed on microalga matrix begins.

The extraction in the second part of extraction curve is according to the BIC model controlled by internal mass transfer resistance *t_i_*, and is dependent on extraction time (*t* = *q*/*q*’). In the model for microalgae extraction, *T*_1_ from Equation (A10) in Appendix is substituted in Equation (24) and we obtain

(25) $$e=x_{u}-\left(x_{u}-e_{1}\right)exp\left(-\frac{K\left(q-q_{1}\right)}{1+\gamma K+\frac{t_{c}}{t_{r}}}\right)\text{ for }q\;>q_{1},\;e\;>\;e_{1}$$

It is evident that the extraction according to this model is also in the second part of extraction curve dependent on the solvent-to-feed ratio *q*.

The approximate relationship between the model parameters determining the second part of extraction curve follows from Equations (23) and (25):

(26) $$t_{i}=\frac{1+\gamma K+\frac{t_{c}}{t_{r}}}{Kq\prime }$$

## 6. Conclusions

The model derived in this study is able to simulate the effects of particle size, flow rate, pressure, and temperature on the mass transfer resistance and the effects of pressure and temperature on the equilibrium parameters solubility, adsorption capacity, and partition coefficient. It is presented in the complete form as Equations (1)–(4), by analytical solution for the lumped-parameter version summarized in Figure 5, and by a simplified, approximate version of the lumped-parameter model in Equations (24) and (25).

The results of modeling the rate of lipid extraction from microalgae with scCO_2_ show the importance of lipid adsorption on the biomass matrix. According to the model, it is the main factor slowing down the supercritical fluid extraction from microalgae, compared to the extraction of vegetable oils where the adsorption is much weaker. The results show that adsorption capacity decreases with increasing pressure and, above the cross-over region, also with increasing temperature. Thus, the extractors for microalgae extraction with scCO_2_ should be designed for substantially higher pressures than 30 MPa, which was regarded as sufficient for the extraction of vegetable oils.

The model assumes that the amount of extractable solute depends on the percentage of microalgae cells opened by the pretreatment and that the solute from closed cells is not available for the extraction with scCO_2_. The only indication that another percentage of cells could be opened later, by exposure to scCO_2_, was found in the work of Solana *et al.* [20], however, as the solvent was modified with ethanol, it could be an effect of the modifier. From the data we had at our disposal we could not recognize whether also neat CO_2_ could open closed cells of microalgae at very high pressures.

Knowledge of characteristics of extraction bed, ε and γ, is necessary for correct estimation of model parameters. Though the number of the present model parameters is as low as possible, the number of parameters adjusted to fit experimental data would have to be even lower to avoid their interaction when fitting experimental extraction yields plotted against time or solvent-to-feed ratio. A separate measurement of extraction curves at large residence times when practically saturated solution flows out of the extractor is therefore recommended. Equilibrium parameters could be determined from this experiment and fixed when the other model parameters, mass transfer resistance and number of mixers, are estimated from experimental runs conducted at higher flow rates.

The modelled data on extraction kinetics support our hypothesis that the low extraction rate, characteristic for the extraction of lipids from microalgae with scCO_2_, is a result of strong oil-matrix interaction. However, though no contradiction against the hypothesis has been found, more extraction experiments examining the effect of varying residence time on extraction kinetics will be necessary to prove it.

## Figures and Tables

**Table 1:** Initial slopes of extraction curves of lipids extracted from crushed and whole aggregates of dry *Chlorella vulgaris* [7] compared with vegetable oil solubility in scCO_2_ calculated according to del Valle *et al.* [22].

Pressure, MPa	Temperature, °C	Slope, g·kg^−1^		Solubility, g·kg^−1^
		Crushed	Whole Aggreg	
20	40	3.1	1.3	3.6
35	40	9.0	2.8	10.4
20	55	1.2	1.3	2.21
35	55	10.8	4.6	11.2

**Table 2:** Model parameters for the extraction of oil from *Nannochloropsis* species [11] (*N* = 1.25 g, *x_u_* = 0.345 kg·kg^−1^, *t_r_* = 2.6–4.7 min) and hydrocarbons from *Botryococcus braunii* [6] (*N* = 2 g, *x_u_* = 0.076 kg·kg^−1^, *t_r_* = 1.2–1.5 min). Flow pattern: ideal mixer.

Ref.	Pressure, MPa	Temp., °C	Flow Rate, g·min^−1^	*y_s_*, kg·kg^−1^	*x_t_*, kg·kg^−1^	*K*, kg·kg^−1^	*t_c_*, min
11	12.5	40	0.35	0.0015	0.25	0.0018	0.45
	20	40	0.35	0.0044	0.11	0.015	0.45
	30	40	0.35	0.0093	0.11	0.049	0.45
	30	40	0.62	0.0093	0.11	0.049	0.30
	30	60	0.35	0.0102	0.08	0.060	0.45
6	12.5	40	0.21	0.0017	0.037	0.018	1
	20	40	0.21	0.0050	0.028	0.030	1
	30	40	0.21	0.0088	0.006	- ^1^	1

^1^ The adsorbed amount was so small that *K* could not be evaluated from the data.

**Table 3:** Model parameters for the extraction of lipids from *Nannochloropsis* sp. [13] (*N* = 180 g, *x_u_* = 0.255 kg·kg^−1^, *t_r_* = 0.8–0.9 min) and *Spirulina platensis* [14] (*N* = 180 g, *x_u_* = 0.078 kg·kg^−1^, *t_r_* = 0.4–0.5 min). Flow pattern: 5 mixers.

Ref.	Pressure, MPa	Temp., °C	*y_s_* ^1^ kg·kg^−1^	*x_t_*, kg·kg^−1^	*K*, kg·kg^−1^	*t_c_*, min
13	40	40	-	>0.255	0.034	2
	55	40	0.019	0.12	0.14	2
	70	40	0.024	0.04	- ^2^	3.5
	40	55	-	>0.255	0.043	2
	55	55	0.026	0.11	0.15	4
	77	55	0.036	0.10	0.28	4.6
14	25	40	-	>0.078	0.026	2
	40	40	-	>0.078	0.075	3
	55	40	-	>0.078	0.17	4
	70	40	0.024	0.028	0.25	5
	25	55	-	>0.078	0.14	2
	40	55	-	>0.078	0.065	3
	55	55	0.026	0.05	0.28	4
	70	55	0.036	0.011	- ^2^	5

^1^ Calculated from the correlation for vegetable oils [22]; ^2^ The adsorbed amount was so small that *K* could not be evaluated from the data.

**Table 4:** Model parameters for the extraction of lipids from *Chlorella vulgaris* [7] (*N* = 5 g, *x_u_* = 0.051 kg·kg^−1^ for whole particles and *x_u_* = 0.125 kg·kg^−1^ for crushed alga, *t_r_* = 2.8–3.5 min). Flow pattern: five mixers.

Particles	Pressure, MPa	Temp., °C	*y_s_* ^1^ kg·kg^−1^	*x_t_*, kg·kg^−1^	*K*, kg·kg^−1^	*t_c_*, min
Whole	20	40	0.0036	0.030	0.01	7
	35	40	0.0105	0.023	0.02	10
	20	55	0.0022	0.022	0.02	2.3
	35	55	0.0112	0.024	0.08	5
Crushed	20	40	0.0036	0.075	0.014	1
	35	40	0.0105	0.07	0.035	1
	20	55	0.0022	0.10	0.010	3
	35	55	0.0112	0.06	0.050	1

^1^ Calculated from the correlation for vegetable oils [22].

**Table 5:** Model parameters for the extraction of lipids from *Nannochloropsis oculata* [30] (*t_r_* = 2.8–3.5 min). Flow pattern: five mixers.

N, kg	*d_p_*, *mm*	*x_u_*, kg·kg^−1^	Pressure, MPa	Temp., °C	*y_s_* ^1^ kg·kg^−1^	*x_t_*, kg·kg^−1^	*K*, kg·kg^−1^	*t_c_*, min
0.010	<0.160	0.125	40	60	0.022	0.085	0.04	0.8
0.010	0.315–1.0	0.105	40	60	0.022	0.085	0.04	0.8
0.010 ^2^	0.16–0.315	0.30	40	60	0.022	0.01	0.04	0.6
0.010 ^3^	0.16–0.315	0.30	40	60	0.022	0.12	0.04	1.3
2.00	0.5	0.20	33	60	0.015	0.03	0.03	1.8
2.00	2.0	0.15	33	60	0.015	0.13	0.05	8

^1^ Calculated from the tripalmitin solubility in scCO_2_ [31]; ^2^ Dried under air flow; ^3^ Freeze-dried.

## Abbreviations

The following abbreviations are used in this manuscript:

LNEG	National Laboratory of Energy and Geology in Lisbon
IST	Instituto Superior Técnico in Lisbon
scCO_2_	supercritical carbon dioxide

## Appendix: Desorption Model

Mass balance equations of the lumped parameter desorption model with two parameters, partition coefficient *K* and fluid mass transfer resistance *t_c_*, are:

(A1) $$\frac{dy}{dt}=-\frac{y}{t_{r}}+\frac{Kx-y}{t_{c}}$$

(A2) $$\frac{dx}{dt}=-\gamma \frac{Kx-y}{t_{c}}$$

(A3) $$e=q\prime \int_{0}^{t}ydt$$

and the initial conditions are:

(A4) $$x=x_{0};\;y=\;y_{0};\;e=0\text{ for }t=0$$

The continuous flow is characterized by specific flow rate *q*’ and residence time *t_r_* (γ = *q*’*t_r_*). The mass balance equations were solved using the Laplace transform with the result:

(A5) $$y=\frac{1}{R}\left(\frac{K\left(x_{0}+\gamma y_{0}\right)}{t_{c}}A+y_{0}B\right)$$

(A6) $$x=\frac{1}{R}\left[\left(\frac{x_{0}}{t_{r}}+\frac{x_{0}+\gamma y_{0}}{t_{c}}\right)A+x_{0}B\right]$$

(A7) $$e=\left(x_{0}+\gamma y_{0}\right)\left[1-\frac{B}{R}-\frac{A}{R}\frac{1+\gamma K}{t_{c}}\right]-\frac{A}{R}\frac{x_{0}}{t_{r}}$$

where

(A8) $$\begin{array}{c} R=\sqrt{S^{2}-4\frac{\gamma K}{t_{c}t_{r}}},\;S=\frac{1}{t_{r}}+\frac{1+\gamma K}{t_{c}},\;p_{1}=\frac{-S+R}{2},\;p_{2}=\frac{-S-R}{2},\;\\ A=\exp\left(p_{1}t\right)-exp\left(p_{2}t\right),\;B=p_{1}\exp\left(p_{1}t\right)-p_{2}exp\left(p_{2}t\right) \end{array}$$

Time constants of the process are *T*_1_ = −1/*p*_1_, *T*_2_ = −1/*p*_2_. The larger of the time constants, *T*_1_, affects the shape of extraction curve to a larger extent than *T*_2_. To express the dependence of time constant on model parameters more transparently, though approximately, the first two terms of Maclaurin series were used to approximate *R*:

(A9) $$R≅S-\frac{2\gamma K}{St_{c}t_{r}}$$

After substitution of Equation (A9) in the expression for *p*_1_, Equation (A8), we obtain:

(A10) $$T_{1}≅\frac{St_{c}t_{r}}{\gamma K}=\frac{t_{c}}{\gamma K}+t_{r}\left(1+\frac{1}{\gamma K}\right)$$

## Notation

*a*_0_	specific surface, m^2^·m^−3^
*A*	function of time in desorption model
*B*	function of time in desorption model
*d_p_*	particle size, m
*D*_12_	binary diffusion coefficient, m^2^·s^−1^
*e*	extraction yield, kg (kg biomass)^−1^
*k_f_*	external mass transfer coefficient, m s^−1^
*K*	partition coefficient, kg biomass (kg CO_2_)^−1^
*M*	CO_2_ in extraction bed, kg
*n*	number of mixers
*N*	biomass feed, kg
*p*_1,2_	roots of the desorption model, s^−1^
*P*	pressure, MPa
*q*	(=*q*’*t*), solvent-to-feed, kg CO_2_ (kg biomass)^−1^
*q*’	(=*Q*’/*N*), specific flow rate, kg CO_2_ (kg biomass)^−1^·s^−1^
*Q*’	flow rate, kg·s^−1^
*R*	constant in desorption model
Re	(=*Q*’*d_p_*/(μ*S_E_*)), Reynolds number
*S*	constant in desorption model
*S_E_*	cross section of extractor, m^2^
Sc	(=μ/(*D*_12_ρ*_f_*), Schmidt number
Sh	(*k_f_d_p_*/*D*_12_), Sherwood number
*t*	time, s
*t_c_*	(=*t_f_* + *t_i_*), characteristic mass transfer resistance time, s
*t_f_*	(=ε/(*k_f_a*_0_)), external mass transfer resistance, s
*t_i_*	mass transfer resistance in particle pores, s
*t_r_*	(=*M*/*Q*’), residence time, s
*T*	temperature, °C
*T*_1_	estimate of time constant in desorption model, s
*V*	volume of extraction bed, m^3^
*x*	solid phase concentration, kg (kg biomass)^−1^
*x_t_*	transition solid phase concentration, kg (kg biomass)^−1^
*x_u_*	(=x_0_ + γ *y*_0_), content of extractable lipids in biomass, kg (kg biomass)^−1^
*y*	fluid phase concentration, kg (kg CO_2_)^−1^
*y_s_*	solubility, kg (kg CO_2_)^−1^
*z*	dimensionless axial co-ordinate
Greek letters	
γ	(=*M*/*N* = *q*’*t_r_*), CO_2_ in extraction bed-to-feed ratio, kg kg^−1^
ε	void fraction
μ	viscosity, kg·m^−1^·s^−1^
ρ*_f_*	CO_2_ density, kg·m^−3^
ρ*_s_*	density of particles, kg·m^−3^
Subscripts	
∞	asymptotic value
+	equilibrium
0-Jan-1900	initial value
1-Jan-1900	end of extraction of free solute
2-Jan-1900	start of desorption
*a*,*b*	two sets of adjusted model parameters
*j*	order of mixer
